# Policy content analysis: Qualitative method for analyzing sub-national insect pollinator legislation

**DOI:** 10.1016/j.mex.2020.100787

**Published:** 2020-01-14

**Authors:** Damon M. Hall, Rebecca Steiner

**Affiliations:** a School of Natural Resources and the Department of Biomedical, Biological & Chemical Engineering, University of Missouri, United States; b Saint Louis University, Lewis & Clark Community College, United States

**Keywords:** Qualitative content analysis, Qualitative content analysis, NVivo, Naturalistic inquiry, Biodiversity conservation, Conservation policy, Law, Policy, Environmental policy, Thematic analysis, Cluster analysis, Qualitative data analysis software, Sustainability science

## Abstract

This project examines sub-national legislative policy to identify trends and describe policy innovations for addressing insect pollinator declines. Content analysis is used to describe these policies quantitatively (number of policies and frequency per year) and qualitatively (topic, comparison of policy instruments used). The policies selected constitute a census—not a sample—of policies passed then approved by all US state legislatures and signed by state Governors into law from 2000 to 2017. We used QSR International’s NVivo 10 text-based analytic software to organize and document our close-reading (line-by-line coding) of 109 laws to address insect pollinator population declines. Our analysis blended both conventional (inductive) and directed (deductive) content analysis approaches to reveal the spectrum of new legislative innovations and to describe lawmakers’ evolving view of pollinating insects. Applying proven methods from the health sciences and communication studies can aid large-scale analysis of legal texts.

• Qualitative content analysis of all US state-level laws passed from 2000 to 2017 addressing insect pollinators (N = 109).
• The close-reading analysis mixed both conventional (inductive) and directed (deductive) content analysis approaches to reveal the spectrum of new legislative innovations and to describe evolving views of pollinating insects.
• Compared inductively gathered findings from US policies to global experts’ policy recommendations to evaluate status of conservation policy.

## Graphical abstract

**Figure:**
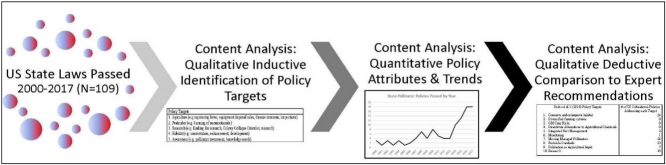

**Specification Table**

**Table:** 

Subject Area:	*Social Sciences*
More specific subject area:	Biodiversity conservation
Method name:	Qualitative content analysis
Name and reference of original method:	Qualitative content analysis See: Berelson, B. 1952. *Content analysis in communication research*. New York, NY, US: Free Press.
Resource availability:	n/a

## Background

Global declines of insect pollinators threaten global food security and economic stability and are due to human behaviors (land uses, habitat alteration, pesticides, etc.) [1,2]. Environmental laws are mutually agreed-upon limits to human behaviors designed to ensure the quality and continuity of shared natural resources [3,28]. In the absence of comprehensive international agreements targeting insect pollinator declines [4], sub-national assemblies are leading the development of policy innovations [5].

We catalog and analyze all pollinator-relevant laws passed by 50 US state legislatures from 2000 to 2017. For a proposed “bill” to become a state law, it must be passed by both legislative chambers (except in Nebraska’s unicameral legislature), then approved “signed” by the state’s elected Governor (executive branch). This study omits all proposed bills and only examines bills that have been passed successfully by legislatures and approved by states’ Governors as law. We use law and policy interchangeably. The study timeframe selected captures before, during, and after widely publicized pollinator declines of the mid-2000’s following the naming of Colony Collapse Disorder, CCD (∼2005), related phenomena affecting native bees (Oregon bee kills 2013), the formation of the Intergovernmental Platform on Biodiversity and Ecosystem Services [2] and its pollinator status report (2016), as well as pre- and post-policy innovations surrounding the US President Obama’s Pollinator Health Task Force (2014). No comprehensive list of these US state-level pollinator policies is readily available—making the policy innovations developed at regional and US state levels less transferable.

Legislation articulates public concerns. This census of policies is meaningful because it documents areas of consensus within sub-national legislative discourse on insect pollinators. US State legislatures reflect the values, opinions, and desires of the populations they represent; agreement requires interactions among rural and urban representatives [6]. Consequently, policies that emerge from these interactions reflect consensus values, cooperation, and agreement that cross traditional party-line and demographic divides such as right-left, rural-urban, rich-poor, religious-secular, etc. These policies also resonate with economic, scientific, cultural, and legal sectors constituting tractable routes for sustainable solutions [7]. This study only examines one subnational body of lawmakers within the US. While this study constitutes a complete account of the activities of state legislators, we do not analyze the lawmaking activities of other branches of subnational governments such as activities of the state governors’ offices or municipal policies (for a full description, see [5]).

The building blocks of law are language. To more closely examine topics of public concern, areas of consensus, and policy innovations relevant to insect pollinators, we use qualitative content analysis to examine the legal actions (content) of recent state legislation. We document policy innovations and characterize policy trends for policy interveners. This empirical account enables lawmaking communities to anticipate and improve lateral and vertical transferability of insect pollinator conservation policy.

## Method details

**Fig. 1:**
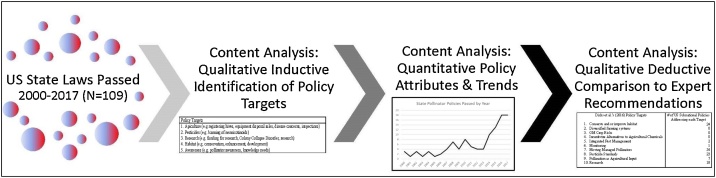
Research tasks.

Qualitative content analysis is not a new approach to textual analysis [8]. This method borrowed from the social and health sciences has only recently been applied to environmental policy topics. Rarely has this method been used to analyze legal documents [9]. Within the environmental fields, qualitative content analysis has been used to analyze environmental policy topics via expert interviews [10], technical reports [11], and newspaper articles on policy [12], however we have found no studies using qualitative content analysis to examine environmental laws or policy texts. This paper outlines our approach to gathering a census of subnational laws from 50 jurisdictions (US states) and using qualitative content analysis to characterize policy trends and showcase the evolution of legal thought on insect pollinator conservation (Fig. 1).

Our approach involved four sets of tasks:

1 Gathering sub-national policies from 2000 to 2017 to ensure a census of laws (texts)
2 Content analysis: Identifying the spectrum of policy targets (themes) via qualitative inductive (conventional content analysis) reading of texts
3 Content analysis: Describing quantitative policy attributes and trends
4 Content analysis: Evaluating the spectrum of themes by qualitative deductive (directed content analysis) comparison to expert recommendations

### Gathering sub-national policies from 2000 to 2017 to ensure a census of laws

Insect pollinator policy at international and national levels suffers from consensus on substance rather than public desire. For example, from 2000 to 2017 the US Senate proposed 31 policies (evidencing issue salience) and passed only four (evidencing intractability). Characterizing the emergence and trends among sub-national insect pollinator policy innovations requires examining a census of all policies within a specified time period, not a sampled selection of policies.

To get a comprehensive set of policies, we searched for policies passed by US state and territories’ legislative bodies between 2000–2017 using usa.gov and the following terms, stemmed words, and Boolean searches: “pollinator AND policy,” “state policy AND pollina*,” “pollination,” “neonicotinoids,” “pesticides,” “colony collapse disorder,” “beekeeping,” “honeybee,” and “honey bee.” After initial results, we expanded the search to combine each states’ name and each search term (e.g., Illinois AND pollinator). Because of limited search results, we searched each US state’s government legislature website (from AL to WY) using the same above keyword combinations. US states’ legislative databases and search engines vary widely. Some state websites limited searching to one year at a time and others prohibited Boolean searches and/or stemmed-word searches. Some state legislative databases only allow a year-by-year search, not a cumulative search. Additionally, some states combine proposed legislation with passed legislation, resulting in additional research to verify bill status.

Because of the variation in states’ legislative databases and lack of results for pollinator policies in 14 states (AK, AZ, AR, FL, GA, IA, MS, NC, ND, OK, SC, SD, TX, WV), we contacted each state directly to triangulate our data. We emailed each state’s legislative librarian requesting “pieces of legislation, including date passed, that pertain to pollinators which could include habitat, importance or awareness of bees and pollinators, pesticides and neonicotinoids, research, and apiculture.” Responses varied. Some answered directly. The majority referred us to the state public librarian, to whom we re-sent the inquiry. We received answers from 42 states (no responses from AR, GA, LA, MS, MO, PA, SD, WV). For the eight states with no response, we repeated data searches on legislative websites and again found no new results.

We found 109 passed laws (see [5]: Table 1). The full texts of the passed, approved, and signed laws constitute the content analyzed.

### Qualitative content analysis: inductive identification of policy targets

We conducted a qualitative content analysis to analyze 1052 pages of text from the 109 laws passed from 2000 to 2017. Content analysis describes a family of approaches for systematic examining of texts [13]. Qualitative content analysis is the close, comprehensive, and organized reading of a set of texts to identify themes, intent, or patterns [14,15]. It is not a mere counting of words but a close reading of texts based on driving research questions [16]. Analysts read a purposeful selection of texts to identify themes or coding frames [17] which are the primary instruments to sort qualitative texts into categories. All the texts are read line by line by the analyst(s) for comprehension. Analysts select relevant lines of text to be “coded” or sorted into themes for comparative study according to research interests. Early uses of content analysis are from fields of psychology, medical practice, and communication research [8,18]. The analysis is grounded in empirical content rather than interpretive argument [9,19].

We used QSR’s NVivo 10.0, a software package for text-based analysis, to store policy texts and to organize our systematic reading. Our objective was to capture and describe the most complete spectrum of policy activities related to insect pollinators. To accomplish this, all laws were read in their entirety line-by-line. The software did not perform any automated functions. The authors read the texts looking for the actions (human behaviors) called for by the laws and responsible actors (government agencies, industry, hobbyists) named in the law. Following conventional qualitative content analysis approach, we had no predetermined codes or themes. Under this “open coding” or inductive approach, all of the coded policy actions fit into 18 thematic categories. Reflection brought this number to a manageable 12 themes [20]. Through discussing and rereading the texts, the authors refined the dozen themes into five parent categories of targeted human behavior (Box 1). Each policy was re-read in its entirety and coded to thematic category for further analysis by one analyst [21].

Box 1 **Insect pollinator relevant policy targets identified via inductive qualitative content analysis.**

Policy Targets

1 Apiculture (e.g., registering hives, equipment disposal rules, disease concerns, inspections)
2 Pesticides (e.g., banning of neonicotinoids)
3 Research (e.g., funding for research, Colony Collapse Disorder, research)
4 Habitat (e.g., conservation, enhancement, development)
5 Awareness (e.g., pollinator awareness, knowledge needs)

Alt-text: Box 1

### Quantitative content analysis of policy attributes and trends

During the search process, we created Table 1 to organize passed legislation by state, including information of title, date passed, and the primary category of law: Apiculture, Pesticides and Neonicotinoids, Habitat, Research, and Awareness (of pollinators). This served as our primary tool for describing the entire body of laws passed. We visualized pollinator policy trends in a line graph by plotting each law and the year it was passed.

**Table 1:** Deductive qualitative content analysis sorting results of the 109 policies that address Dicks et al.’s (2016) policy recommendations/targets for insect pollinator conservation.

Recommended Policy Targets	# of Laws that address each theme	# of references to each theme across all laws
Conserve and or improve habitat	24	38
Diversified farming systems	0	0
GM crop risks	0	0
Incentivize alternatives to agricultural chemicals	0	0
Integrated pest management	2	3
Monitoring	1	1
Moving managed pollinators	54	96
Pesticide standards	23	48
Pollination as agricultural input	7	11
Research	18	22

### Qualitative content analysis: deductive comparison to expert recommendations

We wanted a means to evaluate the US subnational policies in light of other international policy efforts and conversations. To evaluate the spectrum of policies, we identified insect pollinator policy recommendations within the existing literature [4,22,23]. We used NVivo to organize a second deductive line-by-line reading of all of the policy texts [24]. We again read all of the laws in their entirety coding references to policy actions that fit within ten categories identified by [23] “Ten Policies for Pollinators” as a means to characterize the type of legislative action in light of Dicks’ et al. [23] recommendations (Box 2, Table 1). This deductive or systematic reading or “directed” content analysis approach [25] provided an alternative perspective to simultaneously reflect upon the validity of our findings and to evaluate these sub-national policy innovations respective of the experts’ recommendations.

Box 2 **Ten policies for pollinators from Dicks et al. [23].**

1 Raise pesticide regulatory standards.
2 Promote integrated pest management (IPM).
3 Include indirect and sublethal effects in GM crop risk assessments.
4 Regulate movement of managed pollinators.
5 Develop incentives, such as insurance schemes, to help farmers benefit from ecosystem services instead of agrochemicals.
6 Recognize pollination as an agricultural input in extension services.
7 Support diversified farming systems.
8 Conserve and restore “green infrastructure” (a network of habitats that pollinators can move between) in agricultural and urban landscapes.
9 Develop long-term monitoring of pollinators and pollination.
10 Fund participatory research on improving yields in organic, diversified, and ecologically intensified farming.

Alt-text: Box 2

Table 1 shows the results of this deductive rereading of these 109 laws. The number of policies that categorically address Dicks et al.’s [23] ten policy recommendations s were coded under each theme across all policies. The total number of references refer to the number of coded policy actions from all of the policies that fit a category. The number is tracked by NVivo software and reflects how statements were sorted (coded) into a theme during the final close reading of the laws.

## Conclusion

Content analysis of policy texts can identify active discourse among decision making bodies. Our analysis contained both conventional (inductive) and directed (deductive) content analysis approaches to reveal the spectrum, magnitude, and frequency of US state-level laws passed in 2000–2017 addressing insect pollinators. Our analyses reveal the content of the topics, how the insect pollinator matters are framed, and the general trends in policy innovations on this topic.

Following sustainability science’s call to make knowledge usable [26,27], studies like this subnational policy census aim to document policy innovations and characterize trends for policy interveners. This empirical account enables research and lawmaking communities to anticipate and improve lateral and vertical transferability of insect pollinator conservation policy.

## Declaration of Competing Interest

The authors declare that they have no known competing financial interests or personal relationships that could have appeared to influence the work reported in this paper.
